# Established and Emerging Less Invasive Biomarkers and Technologies for Lung Cancer Screening: Puerto Rican Context

**DOI:** 10.3390/onco6020018

**Published:** 2026-04-01

**Authors:** Keisy Rodriguez-Villafañe, Clara Santiago, Juan E. Figueroa, Edwin Figueroa, Yamixa Delgado

**Affiliations:** 1 Pharmaceutical Sciences Department, School of Pharmacy, University of Puerto Rico, Medical Sciences Campus, San Juan, PR 00936, USA;; 2 School of Naturopathic Medicine, Universidad Ana G. Mendez Gurabo Campus, Gurabo, PR 00778, USA;; 3 Biology Department, University of Puerto Rico, Cayey Campus, Cayey, PR 00736, USA;; 4 Biochemistry & Pharmacology Department, San Juan Bautista School of Medicine, Caguas, PR 00726, USA

**Keywords:** lung cancer, early detection, biomarkers, nitric oxide, CYFRA 21–1, CEA, SCC-Ag, NSE, ProGRP, HE4

## Abstract

**Background/Objectives:**

In Puerto Rico (PR), lung cancer mortality remains high because diagnoses frequently occur at advanced stages. Although low-dose computed tomography (LDCT) lowers lung cancer–specific mortality, this screening is difficult to operationalize locally due to high false-positive rates, radiology capacity constraints, payer limitations, and geographic barriers affecting rural populations.

**Methods:**

We performed a narrative review on the literature from 2001–2026 of established and emerging detection strategies—LDCT; serum biomarkers (CEA, CYFRA-21–1, NSE, ProGRP, SCC-Ag, HE4, Hp, TAAb); breath analysis (FeNO and VOCs); and liquid biopsy (ctDNAs/CTCs/miRNAs). We assessed technical performance, feasibility, and health-system fit in PR and then synthesized these findings into an implementable biomarker-first triage workflow for are.

**Results:**

Multiplex serum panels analyzed with machine learning outperform single markers and TAAb provide high specificity with biological lead time, supporting their use as a triage gateway before LDCT. Breathomics is also feasible at the point of care. Liquid biopsy has modest sensitivity in very-early disease yet provides molecular adjudication for indeterminate nodules. A stepwise pathway—expanded risk assessment, integrated multi-panel testing in primary care, LDCT reserved for biomarker-positive individuals, and liquid biopsy when imaging is inconclusive—can enrich pre-test probability, reduce unnecessary scans, align with capitation, and protect limited radiology capacity.

**Conclusions:**

An integrated, non-invasive, biomarker-first triage model offers a pragmatic, equitable route to earlier lung cancer detection in PR and resource stewardship, while reducing disparities.

## Simple Summary

Lung cancer is the leading cause of cancer deaths in Puerto Rico (PR), largely because most cases are found too late. Although annual low-dose computed tomography (LDCT) is the global screening standard, it is difficult to deploy at scale on the island due to high costs, limited radiology workforce and equipment, insurance and logistical barriers, and travel constraints for rural communities. This review outlines emerging biomarkers (serum protein, tumor-associated autoantibodies, and breath sensors) for early detection and a practical, island-wide biomarker-first triage model to identify who most needs LDCT. Because these tests can be performed in primary-care settings across PR, the pathway is easier to access and prioritizes imaging for those at highest risk. By aligning science with local realities, this approach offers a feasible roadmap to detect lung cancer earlier and reduce disparities for all Puerto Ricans.

## 1. Introduction

Lung cancer is a leading cause of cancer mortality worldwide, and Puerto Rico (PR) is no exception [1]. A primary contributor to its high mortality rate is that the majority of cases are diagnosed in advanced stages, when therapeutic options are limited, and the prognosis is unfavorable [2,3]. PR experiences persistently late-stage lung cancer diagnoses and markedly low screening uptake, despite guideline availability. Recent analyses show that the island-wide adherence to low-dose computed tomography (LDCT) screening remains very low (e.g., eligibility–uptake–adherence data from 2022 indicate that <10% of eligible adults are up-to-date), with structural barriers, such as lack of reimbursement, logistics, and patient transportation/fear, frequently cited [4–6]. These data reinforce the need for alternative, easier-to-deploy screening gateways that can be offered at the point of primary care. While various lung cancer detection strategies are implemented globally, their effectiveness is often constrained by a duality of challenges: the inherent technical limitations of the tests themselves, and the specific socioeconomic and infrastructural contexts in which they are deployed. It is critical to note that while screening tests are essential for early detection, a tissue biopsy remains the only definitive method for a lung cancer diagnosis. This review addresses the challenges of early detection by providing a comprehensive overview of screening strategies, from the traditional standard of LDCT to emergent non-invasive technologies. We first examine the universal challenges of these techniques and then analyze the significant implementation barriers—including access, cost, and logistics—that are particularly pronounced within PR’s unique healthcare infrastructure. Ultimately, this review argues that an integrated, multi-marker, non-invasive panel offers the most viable and effective path to overcome existing healthcare disparities and reduce lung cancer mortality across the Puerto Rican population.

Definitions and Intended Use. In this study, we distinguish three clinical terms: screening, triage, and diagnostic. Screening aims to identify individuals at increased likelihood of disease in an ostensibly asymptomatic population; screening tests prioritize high sensitivity to minimize missed cases and acceptable specificity to limit unnecessary follow-up. Triage helps to prioritize who should undergo screening and definitive testing to optimize resource use and reduce unnecessary procedures. Diagnostic use seeks to confirm or exclude disease in a clinical context with higher pretest probability; diagnostic tests emphasize high specificity (and likelihood ratios) to support clinical decision-making.

## 2. Methods for Structured Search

We conducted a structured, narrative review to synthesize established and emerging approaches for earlier lung cancer detection, with an emphasis on feasibility in PR. Literature searches were run in MEDLINE/PubMed, Embase, Scopus and Web of Science. Searches included studies from 2001 to 2026. To capture local implementation and equity considerations, we screened PR’s relevant reports and journals cited by the included articles. We combined controlled vocabulary and free-text terms for lung cancer and screening/diagnostics with modality-specific terms. For example: “lung neoplasms” OR “lung cancer” AND (“screening” OR “early detection” OR “risk stratification”); LDCT terms; serum biomarkers (CEA, CYFRA 21–1, SCC-Ag, NSE, ProGRP, HE4, haptoglobin, “tumor-associated autoantibodies”); breathomics (“fractional exhaled nitric oxide”, FeNO, “volatile organic compounds”, VOCs, “electronic nose”); and liquid biopsy (ctDNA, CTCs, microRNA, methylation). We used citation chaining (backward/forward) from key trials, meta-analyses, and validation cohorts to identify additional records. We included human, adult studies that reported clinical outcomes (e.g., mortality, stage shift), diagnostic performance (e.g., sensitivity/specificity/area under the curve (AUC)), health–economic findings, or implementation data relevant to screening/triage/diagnosis. We excluded editorials, narrative opinions without data, single-case reports, animal/in vitro studies, and studies not specific to lung cancer. We prioritized: (1) randomized controlled trials and systematic reviews/meta-analyses (e.g., LDCT trials and TAAb-triage RCTs); (2) prospective and/or externally validated diagnostic-accuracy studies and large cohort studies; (3) health–economic evaluations and implementation studies pertinent to constrained-resource settings; and (4) emerging technologies validation cohorts were favored over small case–control series to limit spectrum bias.

## 3. Screening Modalities

### 3.1. Established Global Standard: Low-Dose Computed Tomography (LDCT)

LDCT is the established global standard for lung cancer screening in high-risk individuals. Its worldwide adoption was prompted by robust evidence from large, randomized trials such as the National Lung Screening Trial (NLST) in the United States (US) and the NELSON trial in Europe.

#### 3.1.1. Efficacy and Mortality Reduction

LDCT screening results in a significant reduction in lung cancer mortality. The NLST showed a 20% reduction in lung cancer-specific deaths compared to chest radiography [7–9], while the reduction in all-cause mortality was more modest at 6.7% [10]. The primary benefit lies in detecting cancers at an early, more treatable stage, with some studies reporting over 75% five-year survival for stage 1A disease [11]. However, LDCT screening accuracy should not be interpreted as a single fixed value across all screening settings. In a systematic review and meta-analysis focused primarily on baseline population-based LDCT screening studies, Guo et al. reported a pooled AUC of 0.98, a sensitivity of 0.97, and a specificity of 0.87. These pooled values should, therefore, be interpreted in the context of baseline screening and may vary according to the definition of a positive screen, analytic approach, and follow-up framework [12].

#### 3.1.2. Clinical Implementation and Guidelines

International guidelines from bodies, like the US Preventive Services Task Force (USPSTF) and the American Cancer Society, recommend annual LDCT screening for high-risk individuals (typically aged 55–80 with a significant smoking history) [7,13]. However, adherence is suboptimal globally, with screening uptake rates often below 20%, even in countries with established programs [14–16]. Surveys of physicians, including pulmonologists and radiologists, reveal varying opinions and practices regarding screening, which can also impact implementation [15,17,18].

#### 3.1.3. Universal Technical Limitations and Harms

LDCT has several inherent limitations. Although its high sensitivity supports early detection, it also contributes to a substantial false-positive burden, with many initially positive screens ultimately proving to be non-malignant [8,13]. In the NLST, false-positive rates were 26.3% at baseline, 27.2% at year 1, and 15.9% at year 2 [19]. This may cause patient anxiety and lead to unnecessary, and sometimes invasive, follow-up procedures. Across studies reviewed for the USPSTF, false-positive rates ranged from 7.9% to 49.3% at baseline and from 0.6% to 28.6% in incidence rounds, while needle biopsy for false-positive results occurred in 0.09% to 0.56% of all screened participants and surgical procedures in 0.5% to 1.3% [19]. Importantly, the magnitude of false-positive findings depends on the screening protocol, nodule threshold, and definition of a positive examination used across studies and programs. For example, retrospective application of Lung-RADS^®^ to NLST data reduced the false-positive rate from 26.6% to 12.8% at baseline and from 21.8% to 5.3% after baseline, although sensitivity was also lower [20]. Lung-RADS^®^ (Lung CT Screening Reporting and Data System) is a standard of the American College of Radiology for interpreting and reporting findings on LDCT. Overdiagnosis, defined as the detection of slow-growing tumors that may never have become clinically significant, remains another important concern, although reported estimates vary across analyses [8,13]. Additional harms include cumulative radiation exposure from annual scans and the potential for incidental findings that may require further workup [11,13].

#### 3.1.4. The Puerto Rican Context: Lung Cancer Statistics and Amplified Barriers to Implementation

Surveillance data of the Puerto Rico Central Cancer Registry from 2018–2022 indicate that lung and bronchus cancer comprised 5.2% of all newly diagnosed cancers in men and 4.1% in women (ranking third in men and fifth in women), while accounted for 11.6% of cancer deaths in men and 9.3% in women (ranking third) over the same period. The median age for incidence was 72.5 and for death was 75. The survival rate for 5 years in these patients was 21.3%, underscoring the importance of early detection [21]. Although lung cancer is more commonly diagnosed and is the leading cause of cancer death in the US mainland, PR has a higher mortality-to-incidence ratio (~60% vs. 69%), indicating a greater fraction of diagnosed patients who die [22].

Taken together, these surveillance metrics highlight a key contrast of PR and mainland US that motivates a context-specific screening strategy. Importantly, screening uptake remains suboptimal even in the continental US (about 18% of eligible individuals up-to-date in 2022), which underscores that PR’s implementation barriers exist within a broader national challenge but are amplified by island-specific capacity, access, and payer constraints [23].

These disparities are compounded by PR’s status as a US territory (colony), which results in unequal benefits and healthcare funding [5]. The high cost of equipment, lack of specialized personnel, and fragmented healthcare infrastructure make the widespread implementation of LDCT screening unfeasible for most of the island’s high-risk population.

**Figure 1.**
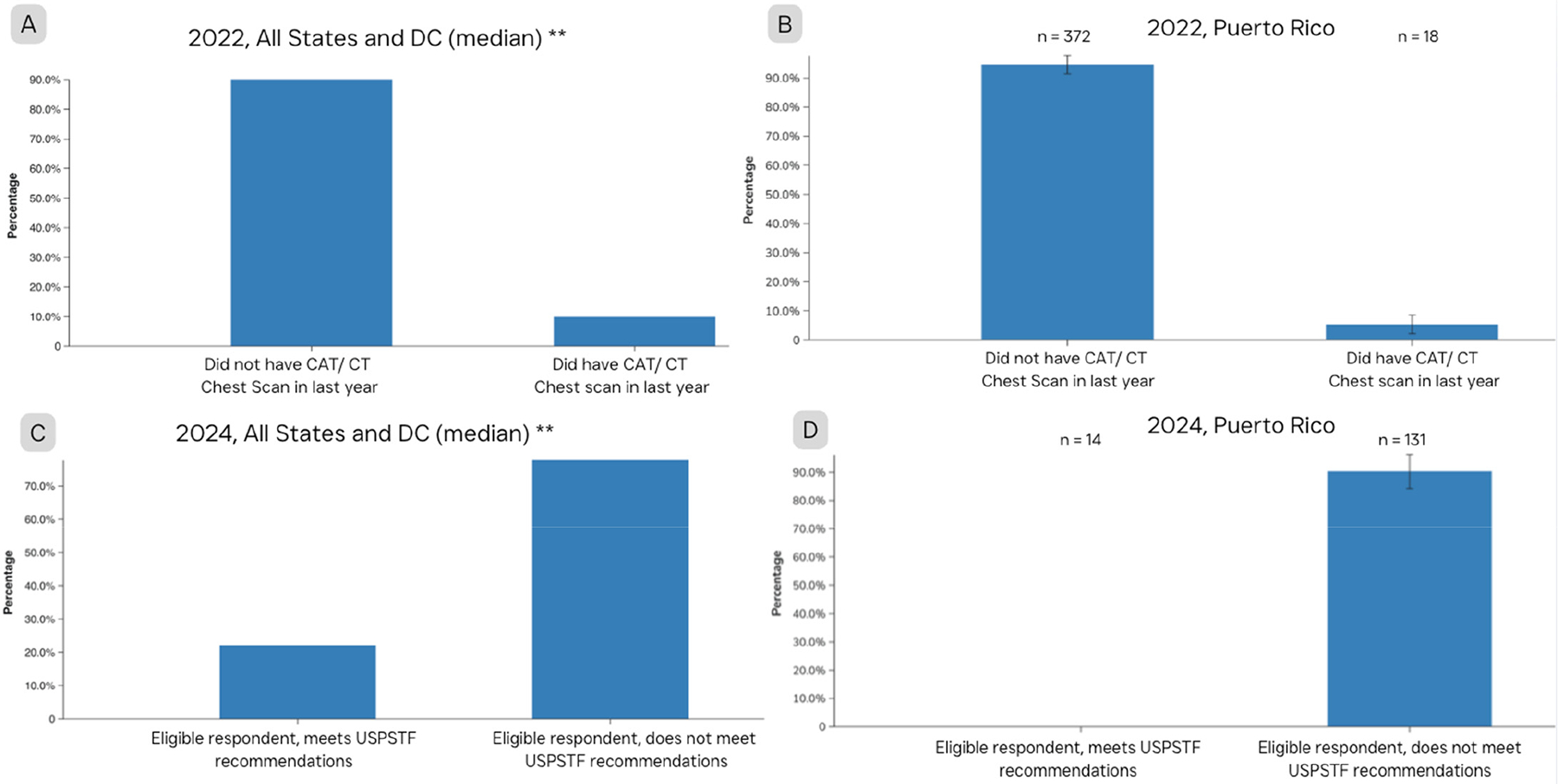
BRFSS Prevalence and Trends Data (2022 and 2024) comparing PR vs. All States and DC (median) for lung cancer screening–related indicators. (**A**,**B**) show self-reported CAT/CT chest scans in the last year for lung cancer screening (2022) for All States/DC (median) and PR; (**C**,**D**) show USPSTF screening eligibility status (2024) for All States/DC (median) and PR. Unweighted sample sizes (*n*) are shown above each bar; estimates with very small denominators (e.g., PR eligible *n* = 14 in 2024) should be interpreted cautiously. Data source: Behavioral Risk Factor Surveillance System (BRFSS). The CT/CAT chest scan serves as a proxy for LDCT screening. ** Median value reported with no confidence intervals [24].

Recent data on screening eligibility and uptake on the island confirm these challenges, highlighting the persistent low adherence rates [6]. Also, population-based surveys reinforce the low screening uptake. In the 2022 BRFSS Prevalence & Trends Data, only 5.4% of PR respondents reported having a CAT/CT chest scan in the prior year for lung cancer screening (vs. 9.9% in the continental US), while 94.6% reported none (vs. 90.1% in the continental US). This disparity persists in 2024 (90.4% PR vs. 77.9% continental US), and the BRFSS eligibility module suggests that screening-eligible respondents in PR are exceedingly few (unweighted *n* = 14), indicating that BRFSS captured very few screening-eligible respondents in that survey cycle and reinforcing how uncommon screening exposure appears in population surveys (Figure 1) [24]. In another study by Castañeda-Avila (2025), island-specific estimates suggest that about 94,955 PR residents in 2022 met the criteria for screening with LDCT, representing about 7.9% of the population. However, only 9.8% were up to date with screening, which is lower than mainland US Hispanic (17.3%) and non-Hispanic residents (18.1%) [6]. This pattern suggests persistent implementation and adherence gaps, despite eligibility. These findings are particularly striking given that the Puerto Rico Comprehensive Cancer Control Plan explicitly targets increased utilization of LDCT/CAT/CT screening from 5.4% in 2022 to 7.5% by 2030 [25]. However, the BRFSS trend suggests that implementation has not yet translated into measurable population-level uptake. Moreover, a PR non-small cell lung carcinoma (NSCLC) real-world cohort reported that 71.1% of patients were diagnosed at stage III–IV, consistent with delayed detection and reinforcing the need for earlier detection pathways that are feasible under local constraints [26]. A plausible contributor to this gap is that policy adoption alone is insufficient: screening requires operational implementation by payers and health systems, including coverage determination, prior authorization rules, reimbursement pathways, and network capacity—steps that can substantially delay or limit real-world delivery, even when screening is recommended. Consistent with these patterns, no comprehensive lung cancer screening program currently operates island-wide in PR. In a recent survey, 77% of pulmonologists reported obstacles to performing LDCT screening, citing medical insurance plan requirements and logistical challenges as the most frequent impediments [27].

Geographic limitations likely contribute to constraints in LDCT uptake. PR is predominantly urban (91.9%); however, a meaningful minority of residents live in rural areas (8.1%), where distances to imaging sites, transportation, and specialist availability can increase friction across the multistep screening pathway [28]. These factors can also interact with participation barriers, along with fear, low health literacy, lack of insurance coverage, and limited referral continuity influencing screening implementation [4,5]. Likewise, Rodríguez-Cintrón et al. 2022, reported that many pulmonologists did not have access to chest CT services within 1 h of their practices, indicating limited local availability of imaging capacity for screening [27]. In this context, geographic dispersion and travel burdens can disproportionately reduce screening completion, especially among older adults and lower-resource patients, thereby worsening inequities.

In addition, PR faces a sustained radiologist workforce constraint and imaging bottlenecks, which make population-scale LDCT difficult to operationalize without triage. Large-scale clinician emigration has made the physician workforce decline from 14,500 in 2009 to 9000 in 2020 [29]. PR-specific radiologist-per-population estimates and LDCT-capable scanner inventories are not consistently reported in publicly available sources; therefore, we contextualize radiology capacity using the available workforce migration trends. Reports describing the radiologist shortage and global capacity gaps, combined with local physician migration after economic crises and natural disasters, suggest that any island-wide program must first enrich the pre-test probability before imaging [30–32].

A further constraint is that PR’s Government Healthcare Plan (Vital) operates largely on capitation; therefore, programs with high false-positive downstream costs (like LDCT without triage) are difficult to sustain. Documentation from “Administración de Seguros de Salud” (ASES) or (Health Insurance Administration), an agency that manages the government healthcare plan in PR, describes capitation as a core financing mechanism that supports the payer-incentive argument for reducing downstream utilization [33]. Thus, a high-specificity biomarker gateway that reduces negative scans and unnecessary procedures better aligns with payer incentives [34,35].

Importantly, PR’s low uptake likely reflects multiple binding constraints—participation/behavioral factors (transportation, fear, health literacy), payer implementation barriers (coverage/authorization), and imaging workforce/capacity limitations.

### 3.2. Emerging Non-Invasive and Minimally Invasive Technologies

**Figure 2.**
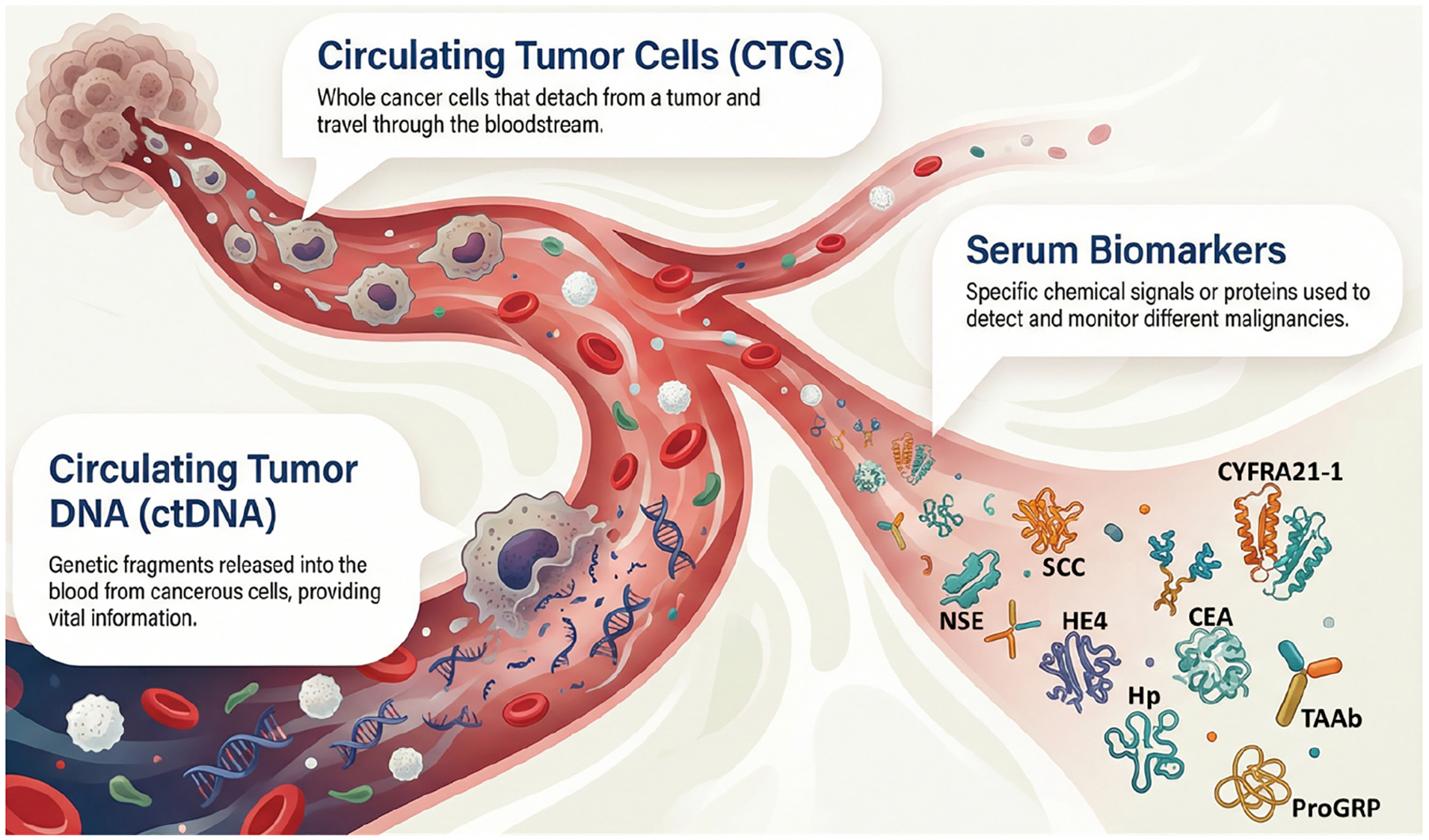
Bloodstream as a reservoir of ctDNA, CTCs and serum biomarkers for the early detection of lung cancer.

The search for alternatives that can overcome the limitations of LDCT has led to the development of numerous non-invasive and minimally invasive technologies. These globally researched strategies focus on detecting biomarkers in accessible biological samples. Figure 2 shows the circulating components (serum biomarkers, circulating tumor DNA (ctDNA) and circulating tumor cells (CTCs)) in the bloodstream relevant for cancer detection and monitoring.

#### 3.2.1. Serum Biomarkers

A biomarker is a defined characteristic—molecular, histologic, radiographic, or physiologic—that is measured as an indicator of normal biological processes, disease processes (such as early, preclinical cancer), or responses to an exposure or intervention. In screening, biomarkers are used to identify individuals at elevated risk in order to enable earlier detection and more effective triage to diagnostic evaluation [36].

##### Carcinoembryonic Antigen (CEA):

CEA is a glycoprotein involved in cell adhesion. While its levels are often elevated in lung cancer, particularly adenocarcinoma, it is not specific to this disease. As a general tumor marker, CEA can also be elevated in colorectal, pancreatic, gastric, and breast cancers, as well as in non-malignant conditions like smoking-related inflammation. Its sensitivity is moderate, with higher levels correlating to advanced stages and metastasis [37]. For example, Li et al., 2024 (case–control study) found that serum CEA levels were significantly higher in patients with NSCLC (*n* = 184) than in those with benign lung disease (*n* = 60) or healthy controls (*n* = 90) (*p* < 0.01) [38]. From the same study, as a single marker, CEA showed modest diagnostic discrimination for NSCLC (AUC = 0.638); however, when integrated with TuM2-PK and CYFRA21–1, accuracy was improved (AUC = 0.918). Furthermore, a retrospective analysis of newly diagnosed lung cancer focusing on metastasis assessment in 213 patients found that serum CEA levels were significantly higher in patients with metastasis (*p* < 0.001, AUC = 0.724), suggesting its utility in identifying advanced disease stages [37]. Based on PR-specific demographics, CEA levels could be explored as a low-cost triage adjunct. Smoking rates are lower in PR (8.5%) than in the mainland US (11.6%) making adenocarcinoma one of the most common types of lung cancer in this population [24,39,40]. Adenocarcinoma can present with elevated CEA levels; thus, this histologic distribution may increase the overall prevalence of this marker. Since smoking may cause false elevations in CEA, the lower smoking prevalence may improve CEA’s specificity in PR patients [39,41].

##### Cytokeratin 19 Fragment (CYFRA21–1):

CYFRA21–1 detects fragments of cytokeratin 19 released from epithelial tumor cells. It is particularly useful in NSCLC, with sensitivity up to 71% and specificity up to 94%, according to a case–control study (newly diagnosed NSCLC vs. similar demographic) [42]. While more specific to NSCLC than CEA, it can also be elevated in other epithelial cancers like bladder cancer. It is especially helpful in detecting squamous cell carcinoma and shows a strong correlation with disease stage [43]. Given PR’s adenocarcinoma-predominant histology and CYFRA21–1’s stronger performance in squamous disease, CYFRA21–1 is best framed as a multi-marker panel component for risk enrichment/triage rather than a stand-alone screening marker, with value for enriching detection of the squamous NSCLC subset [26,44]. A comparative study (three group case–control study of lung cancer vs. benign pulmonary disease vs. healthy) shows that levels of CEA and CYFRA21–1 were significantly higher in lung cancer patients; the combination of these tumor markers along with NSE showed the highest sensitivity, reaching 90% for early detection, outperforming other diagnostic methods [45]. Another study comparing multiple markers (hospital cohort/case–control of malignant vs. benign/healthy) confirmed these findings, where CYFRA21–1 showed the highest sensitivity (70%) for detecting lung cancer [46]. In summary, its performance is best within multi-marker panels [42,47,48].

##### Squamous Cell Carcinoma Antigen (SCC-Ag):

As its name suggests, SCC-Ag is most specific for squamous cell carcinoma. However, this is a cell type, not a cancer type, meaning that the marker can be elevated in squamous cell carcinomas of the lung, cervix, head, and neck. Its sensitivity for lung cancer is lower than other markers, limiting its use as a stand-alone diagnostic tool [49]. In a study by Li (2021) (three-group case–control of malignant vs. benign vs. healthy) involving 120 participants with lung cancer, serum SCC-Ag levels were significantly higher in squamous cell carcinoma compared to adenocarcinoma or small cell lung cancer, reinforcing its specific utility for this histological subtype. However, the study emphasized that a combined panel (with CEA, CYFRA 21–1, and ProGRP) yielded a superior diagnostic sensitivity of 93.29% and a specificity of 84.32%, significantly outperforming the sensitivity of markers used in isolation [50]. In adenocarcinoma-predominant settings, such as PR, SCC-Ag is unlikely to be a strong stand-alone marker; however, it may add incremental value and disease differentiation within multi-marker panels [26].

##### Neuron-Specific Enolase (NSE):

NSE is an enzyme released from neuroendocrine cells and is strongly associated with tumors of this origin, most notably small cell lung cancer (SCLC). Its high specificity for neuroendocrine tumors makes it a valuable tool to help distinguish between SCLC and NSCLC, although its sensitivity is lower for NSCLC. In PR, NSE is best positioned as a subtype-oriented differential adjunct rather than a population-level screening marker. NSE is most effective when combined with ProGRP for SCLC screening [48,51,52]. In a case–control study, a group of researchers found that NSE showed higher sensitivity for small-cell carcinoma (AUC = 0.775) than for squamous carcinoma (AUC = 0.719) [53]. Leveraging real-world data, a retrospective descriptive utilization study showed that within 3 months before first diagnosis, NSE was obtained in ~65% of NSCLC and ~80% of SCLC cases, underscoring its screening role [54].

##### Progastrin-Releasing Peptide (ProGRP):

ProGRP is currently regarded as one of the most accurate and specific biomarkers for SCLC, where it can yield an AUC as high as 0.973 [47,52,55,56]. Its utility for NSCLC is limited. As with NSE, ProGRP is best used to discriminate between the main classifications of lung cancer and to guide physician workups when elevated. In a study of 452 participants (diagnostic cohort/case–control of SCLC vs. NSCLC/benign), Oh et al. (2016) reported that plasma ProGRP reached a sensitivity of 85.7% and a specificity of 90.2% for SCLC, proving also to be a valuable tool for tumor staging and monitoring responses to chemotherapy [57]. In a retrospective cohort of 290 patients with lung neuroendocrine neoplasms, Rosiek et al. (2023) demonstrated that ProGRP is an exceptionally effective diagnostic marker, achieving a sensitivity of 94.8%, a specificity of 100%, and an AUC of 0.995 when distinguishing lung neuroendocrine neoplasms against healthy controls [58].

##### Human Epididymis Protein 4 (HE4):

HE4 is an emerging marker showing promise for lung cancer but its primary clinical use is as a marker for ovarian cancer. In recent studies, HE4 has shown a high AUC for overall lung cancer detection when combined with traditional markers for both subtyping and diagnosis [47,59]. This finding suggests that it can be proposed as an investigational adjunct/panel candidate for screening in PR patients, although validation is needed for this cohort. The combination of HE4 with ProGRP and NSE was seen as a promising and optimal strategy for SCLC [47]. A case–control study by Iwahori et al. in 2012, using a novel ELISA system, evaluated 49 lung cancer patients and found that serum HE4 levels were significantly elevated in both NSCLC and SCLC, with an exceptional AUC = 0.988, a sensitivity of 89.8% and a specificity of 100% [60]. Another case–control study focusing on male lung cancer patients (*n* = 98) found that HE4 levels were significantly elevated compared to healthy controls (*p* < 0.001), with an AUC of 0.848 and a sensitivity of 64.3% at 95.9% specificity [61].

##### Haptoglobin (Hp):

Hp is a type of acidoglycoprotein that has shown high sensitivity to lung cancer, especially when combined with CEA, NSE, and CYFRA21–1. Its positive detection rate is highest in the squamous cell carcinoma subtype [62]. For the PR population, this marker serves a complementary role in distinguishing between the most prevalent types of NSCLC on the island, improving sensitivity for squamous cell carcinoma. In a case–control study utilizing proteomic analysis followed by validation in lung cancer patients, Hp showed an AUC of 0.768 for distinguishing lung adenocarcinoma from healthy controls, with a sensitivity of 64% [63]. Furthermore, a study (three-group case–control of lung cancer vs. benign vs. healthy) involving 193 subjects with lung cancer and 87 with benign disease reported that while serum Hp alone had a moderate sensitivity of 43.5% and a specificity of 90.7%, its combination with CEA, NSE, and CYFRA 21–1 significantly increased the overall positive detection rate to 85.0%, particularly aiding in pathological typing [62].

##### Tumor-Associated Autoantibodies (TAAb):

TAAb arise when the host immune system mounts a humoral response against tumor antigens (e.g., p53, NY-ESO-1, CAGE, GBU4–5, HuD, MAGE-A4, SOX2), providing biological signal amplification that can be detectable months to years before radiologic visibility [64]. For example, a large, pragmatic, randomized, controlled trial (12,208 participants) called the Early Detection of Cancer of the Lung Scotland (ECLS), used the EarlyCDT-Lung autoantibody blood test, offering LDCT only to test-positive participants (every 6 months for up to 2 years), while test-negative and control participants received the usual care [65]. Notably, they observed a stage shift with fewer stage III/IV diagnoses versus usual care (hazard ratio for stage III/IV = 0.64; 95% CI 0.41–0.99), supporting the role of autoantibodies as a high-specificity triage rather than a stand-alone screen. At the same time, independent evaluations reported modest sensitivity for EarlyCDT-Lung (≈30–40% overall and lower for stage I–II) [66]. These findings are relevant for PR, where contemporary cohorts indicate that a high proportion of NSCLC patients present with advanced disease, consistent with documented screening implementation barriers [26]. This proposed biomarker could help to make a more promptly diagnosis in the Puerto Rican population.

#### 3.2.2. Exhaled Breath Analysis

##### Nitric Oxide (NO):

Aberrant NO signaling is a central feature of cancer [67]. The measurement of fractional exhaled nitric oxide (FeNO) is a non-invasive technology that reflects changes in airway inflammation. FeNO is generally elevated in lung cancer patients when compared to healthy controls; its levels correspond to tumor burden and stage, especially in NSCLC [68]. However, FeNO can also be elevated in other inflammatory lung conditions such as asthma. It shows good sensitivity for distinguishing lung cancer patients from controls but may lack adequate clinical specificity if used alone [69]. In PR, asthma and other respiratory diseases are very prevalent due to air pollution; thus, this marker may be insufficient on its own but can serve as an adjunct in a multi-modal approach to lung cancer screening [70,71]. Furthermore, alterations in exhaled NO may also correlate with changes in cytokine profiles, such as IL-17 and VEGF, linking NO production to ongoing tumor-mediated inflammation and angiogenesis [72].

##### Exhaled Breath Analysis (VOCs, Sensor Arrays):

Sensor-based technologies enable the multiplexed detection of volatile organic compounds (VOCs) and other exhaled gases (e.g., NO, acetone, isoprene), providing a rapid and non-invasive strategy for lung cancer screening. Recent advances in electronic nose (“e-nose”) systems with metal oxide sensors have improved both the sensitivity and selectivity of breath-based diagnostics. These platforms hold promise for point-of-care application, especially when incorporated with ensemble learning models for higher diagnostic accuracy [69,73,74]. For patients in PR, a breath-based triage may provide a low-burden primary-care gateway that helps prioritize limited imaging capacity amid workforce constraints, capitation, and payer authorization requirements.

#### 3.2.3. Liquid Biopsy (ctDNA, CTCs, miRNA)

Liquid biopsies detect tumor-derived materials such as ctDNA and CTCs from the blood [75–77]. Recent tumor-informed ctDNA assays with ultrasensitive, phased-variant sequencing achieve subparts-per-million limits of detection and have doubled clinical sensitivity for post-operative minimal residual disease compared with first-generation assays, enabling recurrence prediction before imaging [78,79]. In the indeterminate pulmonary nodule setting, integrating plasma ctDNA methylation with clinical and CT features improved malignant/benign classification, especially for 5–10 mm nodules [80]. CTCs complement ctDNA by offering cellular phenotyping; epithelial/mesenchymal hybrid CTCs correlate with metastatic potential in NSCLC and can aid in subtype discrimination and resistance monitoring when combined with ctDNA in multimodal workflows [81].

As components of liquid biopsy, miRNAs are part of tumor-derived materials that can be detected without a tissue biopsy; alongside ctDNA and CTCs, a liquid biopsy includes circulating RNA, either free in plasma or packaged in extracellular vesicles such as exosomes [82]. In a large multicenter cohort of 3046 individuals, a blood-borne miRNA signature achieved 91.4% accuracy (82.8% sensitivity, 93.5% specificity) for lung cancer detection across symptomatic populations and clinically relevant controls [83], supporting utility for triage and enrichment prior to LDCT and demonstrating disease-focused specificity rather than generic inflammation signals alone. Beyond single analytes, exosomal miRNA panels measured in serum improved analytical robustness and biological specificity (vesicular protection; tumor-derived cargo), with optimized multi-miRNA combinations reporting ROC AUC > 0.93 for early-stage NSCLC in discovery/validation workflows—an attractive feature for pre-imaging screening or parallel testing with serum proteins [84]. Importantly, integrated approaches that combine plasma miRNAs (e.g., miR-21–5p) with signals from other matrices (e.g., sputum miR-31–5p/miR-210–3p) have demonstrated 85.5% sensitivity and 91.7% specificity, independent of stage and histology, reinforcing that miRNA can raise pre-test probability and increase PPV when embedded in multiple, machine learning-assisted panels alongside established serum biomarkers [85]. The aggregate evidence indicates that miRNAs—individually, in vesicles, or in integrated panels—offer lung-relevant specificity and screening performance that can be operationalized within a biomarker-first workflow to streamline LDCT utilization.

While ctDNA and methylation-based assays represent major advances in liquid biopsy, their performance for very-early, asymptomatic disease remains limited. Much of the evidence reporting high sensitivity originates from diagnostic or minimal residual disease (MRD)-oriented cohorts rather than true screening populations, as highlighted in reviews of liquid-biopsy implementation and limitations [75,79]. Moreover, the detection of low-variant-allele-frequency ctDNA can be confounded by clonal hematopoiesis, which produces non-tumor mutations that reduce specificity, particularly in older individuals [79]. This is particularly relevant in PR, given its older age structure (≈24.6% of residents are ≥65 years), which may increase the prevalence of clonal hematopoiesis and necessitates careful assay calibration and external validation in local populations [86,87]. Assay-specific differences in sequencing depth, target panels, bioinformatic pipelines, and analytical sensitivity introduce additional variability across platforms [75]. The high cost of advanced NGS-based ctDNA assays currently limits their feasibility for broad population screening. For these reasons, ctDNA and methylation assays should be interpreted as adjunct tools, with their most validated roles in: (1) the adjudication of indeterminate pulmonary nodules, where integrating plasma methylation with LDCT and protein markers improves malignancy discrimination; and (2) MRD detection and recurrence monitoring, where ultrasensitive tumor-informed approaches have demonstrated strong predictive value [78,80]. At present, no prospective population-screening trials support ctDNA or methylation assays as first-line screening tests; therefore, we position them as complementary technologies rather than primary screening modalities.

Implementation of liquid-biopsy procedures in PR should account for variability in clinician knowledge and the utilization of lung cancer biomarker testing, reinforcing the need for standardized workflows and decision support if deployed [88].

#### 3.2.4. Universal Limitations of Emerging Biomarkers

These emergent technologies share universal technical limitations. A primary challenge is often insufficient sensitivity and specificity for early-stage disease. Many serum markers are also elevated in non-malignant inflammatory conditions, leading to false positives. It is important to highlight that reported diagnostic accuracies for serum biomarkers are often from case–control or symptomatic cohort studies, which could inflate their sensitivity, specificity, and AUC results. This is because the information is from symptomatic cohorts, clinically indicated diagnostic workups, or case–control designs, rather than true asymptomatic screening populations. This means that these settings often include a higher proportion of advanced-stage cancers and fewer benign comparators. This may create spectrum bias, meaning that performance does not generalize to asymptomatic individuals undergoing screening. In addition, in true screening settings with low disease prevalence, even the highly specific biomarkers yield lower positive predictive values (PPV), which increases false-positive referrals. Therefore, all accuracy estimates in this section should be interpreted within the context of each study setting, population, and stage distribution, and not assumed to reflect screening-level performance. Moreover, multi-biomarker panels with modest sample sizes are prone to overfitting when developed by machine learning models. The performance can be inflated by batch effects such as systematic differences in sample handling between cases and controls. Ultimately, many panels lack external validation with independent screening-like cohorts. This makes data preliminary and it may not transport to real-world screening settings.

Exhaled breath analysis is susceptible to confounding variables like diet and coexisting respiratory conditions. Liquid biopsies can struggle to detect the low levels of ctDNA present in early-stage cancer. Finally, many of these technologies are still experimental, costly, and lack the standardization needed for widespread clinical use as screening tools.

Another limitation is that PR-specific validation data for these emerging biomarkers in population-based cohorts is limited. As a result, reported trends are extrapolated from other groups. Differences in population characteristics and workflows may affect calibration and generalizability, underscoring the need for external validation. A study is currently being designed to evaluate these biomarkers directly in PR lung cancer patients and to generate local performance data.

### 3.3. Additional Emerging Technologies

- Genetic/Epigenetic Markers: Assays, such as DNA methylation tests of SHOX2/RASSF1A in bronchoalveolar lavage fluid (BALF), have been shown to provide higher sensitivity than serum CEA in early lung cancer detection and can serve as a diagnostic adjunct when cytology is inconclusive [89];
- Advanced Imaging: Near-infrared fluorescence tumor-targeted imaging offers enhanced intraoperative detection of cancerous nodules, supporting surgical navigation [90];
- Tumor Markers in Other Fluids: Traditional and novel markers can also be assayed on pleural fluid for diagnosing malignancy [91]. Pleural fluid, defined as liquid around the lungs, has emerged as a valuable biological matrix for evaluating lung cancer–associated malignant pleural effusion. Several tumor biomarkers, including CEA, CYFRA-21–1, NSE, SCC-Ag, and ProGRP, have been rigorously studied for their diagnostic performance in this compartment. Among these, CEA consistently demonstrates the highest diagnostic accuracy: a major 2020 study analyzing 348 patients with pleural effusions identified pleural-fluid CEA at a cutoff of 5.23 ng/mL as the most effective marker, achieving 99% sensitivity and 91.6% accuracy in distinguishing malignant from benign effusions, outperforming CYFRA-21–1, SCC-Ag, and NSE [92]. NSE and ProGRP are detectable in pleural fluid but generally yield lower diagnostic performance compared with CEA and CYFRA-21–1; however, they remain clinically useful for differential diagnosis and the histological typing of malignant pleural effusion [93]. SCC-Ag is also measurable in pleural fluid but consistently performs weaker than CEA and CYFRA-21–1 in differentiating malignant from benign effusions [92]. Collectively, these studies show that pleural fluid biomarkers—especially CEA, followed by CYFRA-21–1—provide meaningful diagnostic support in lung cancer evaluation. However, it is important to note that obtaining pleural fluid requires thoracentesis, an invasive medical procedure. For this reason, it is not considered the best matrix for early screening.

Beyond pleural fluid, saliva and sputum offer non-invasive matrices that can capture the same molecular signals discussed for plasma/serum. In saliva, case–control modeling with miRNAs (e.g., let-7a-2, miR-221, miR-20a) achieved up to 90% accuracy (95% sensitivity, 85% specificity) for distinguishing lung cancer from controls, underscoring the feasibility for point-of-care triage in high-risk populations [94]. Complementarily, a salivary metabolomics study that profiled paired tumor/adjacent tissues, plasma, and saliva (*n* ≈ 109 pairs) identified 12 metabolites shared across matrices (polyamine/amino-acid pathways) and produced a 12-metabolite salivary panel with validation AUCs~0.74–0.79, reinforcing saliva as a proxy for early tumor biology [95]. Despite the absence of studies examining the early screening of lung cancer through the detection of the serum protein biomarkers discussed in this review, certain serum markers, including CEA, CYFRA-21–1, NSE, and Hp, have been identified in saliva [94,96–98]. This finding underscores the direct translational potential of serum markers in salivary testing for community-based triage. However, salivary marker studies require strict methodological control, as concentration levels can differ significantly between individuals and are affected by oral health. Therefore, creating standard criteria for normal and pathological ranges is essential for accurate diagnosis.

In sputum, an integrated panel that combines sputum miRNAs (miR-31–5p, miR-210–3p) with plasma miRNAs (miR-21–5p) improved discrimination of NSCLC, with 85.5% sensitivity and 91.7% specificity, independent of stage and histology [85]. Regarding protein biomarkers, Kalomenidis (2004) discovered that CEA, CYFRA21, and NSA attained sensitivities in sputum that were analogous to those observed in the serum of lung cancer patients [99]. In principle, sputum is produced from the respiratory tract, which could improve specificity for early lung cancer screening. Collectively, these saliva/sputum data demonstrate that other fluids can detect biomarkers already used in serum together with miRNA and metabolite signatures, enabling earlier, lung-focused case findings when deployed as machine learning–assisted multiplex panels alongside imaging. For PR, saliva/sputum triage and methylation-based adjudication are plausible for near-term applications. These non-invasive methods may be useful to increase screening capacity before LCDT referral, lowering late-stage detection. Nevertheless, PR-specific validation in local cohorts is necessary before broader adoption.

### 3.4. Future Developments and Ongoing Research

Future directions for LDCT screening aim to optimize eligibility criteria using individualized risk models, minimize harms by refining follow-up protocols, and enhance cost-effectiveness. There is also a push to expand screening to certain non-high-risk populations, such as never-smokers and younger adults, as evidence accumulates [7,69,100].

Table 1 consolidates the core characteristics and performance of the gold standard LDCT, serum biomarkers, breath analytes and liquid biopsy—linking each test/marker to its sample type, invasiveness, targeted lung cancer subtype(s), clinical role/utility and diagnostic value—to enable rapid, evidence-based comparison across options. This harmonized view highlights complementarities among biomarkers and technologies, clarifies where each test adds incremental value, and supports reproducible panel design tailored to histology and care settings.

Table 2 highlights a growing shift toward non-blood, lung-proximal biofluids to improve early lung cancer detection by capturing biomarkers closer to their site of origin. In pleural fluid, traditional tumor markers show strong discriminatory performance in clinically indicated effusions. However, because pleural fluid collection is inherently procedure-dependent and often reflects advanced disease contexts (e.g., malignant pleural effusion), Table 2 motivates future work focused on less invasive respiratory matrices, such as sputum and saliva, for population-relevant screening.

## 4. Discussion

The dual challenge of lung cancer screening—universal technical limitations combined with specific contextual barriers—is starkly evident in PR. While LDCT is the global standard, is not a good fit for an island with limited resources and a fragmented healthcare system. Similarly, while emerging biomarkers are promising, their own technical limitations (e.g., variable sensitivity/specificity) prevent any single one from being a standalone solution.

This is where the promise of a multi-faceted approach becomes clear. An integrated, multi-marker, non-invasive panel offers the most viable path forward for Puerto Rican patients. Single serum markers rarely achieve acceptable screening performance when used alone; combining markers that capture orthogonal biology (e.g., tumor load, neuroendocrine features, epithelial injury) improves accuracy.

The recommended biomarkers for our proposed first-triage panel are CEA + CYFRA-21–1 + NSE + ProGRP + SCC-Ag + HE4. These biomarkers can be analyzed with machine learning classifiers rather than fixed cut-offs, demonstrating substantially higher discrimination for lung cancer, and for small-cell histology, in particular [47,48,56,62,102]. They were selected based on their complementary histology coverage—adenocarcinoma (CEA, HE4), squamous (CYFRA 21–1, SCC-Ag), and SCLC (ProGRP, NSE)—and superior performance in combination versus any single marker, which is critical for a triage-first approach that seeks to raise specificity and contain the imaging burden. In parallel, TAAb offers a high specificity and biological lead time, making TAAb well-suited as a triage gateway before LDCT to limit downstream false-positive imaging [65]. Additionally, integrating a low-cost breath sensor array—FeNO plus VOC profiling via e-nose—into the panel is feasible; e-nose systems have distinguished lung cancer from other pulmonary diseases and are amenable to point-of-care use, especially when locally calibrated for ambient humidity and particulate conditions [69,73,74]. Finally, for indeterminate nodules or borderline-risk cases, a blood-based liquid biopsy (ctDNA/CTCs) can serve as a molecular adjudicator alongside imaging—useful to refine malignancy probability and, in postoperative settings, to detect minimal residual disease—although sensitivity in very early-stage disease remains a limiting factor [35]. In very early-stage settings, a multimodal biomarker panel (serum proteins + tumor autoantibodies ± e-nose) typically achieves higher practical sensitivity than the ctDNA assay, whereas ctDNA contributes molecular specificity and is best positioned as an adjudicator for indeterminate nodules and for recurrence monitoring rather than as a first-line screening replacement [103]. However, consistent with expert guidance emphasizing the importance of study design in biomarker evaluation, we deliberately avoid overstating early-disease sensitivity for ctDNA or methylation assays and instead highlight their current, evidence-supported roles in nodule adjudication and MRD assessment [66].

Hispanic/Latino cohorts—including Puerto Ricans—exhibit higher EGFR mutation prevalence (approaching ~24–40%) and relatively lower KRAS rates compared with non-Hispanic Whites [40,104]. Screening and triage panels for PR should, therefore, ensure the sensitive detection of EGFR (Ex19del/L858R) and consider ALK/ROS1 as part of a liquid-biopsy adjudication step for indeterminate LDCT findings. Building in-island rapid-turnaround workflows (e.g., automated NGS platforms) can shorten the time to result and reduce send-out costs. This type of research also moves medicine toward a more personalized approach, allowing for the development of diagnostic algorithms tailored to the unique genetic and environmental profile of the Puerto Rican population.

**Figure 3.**
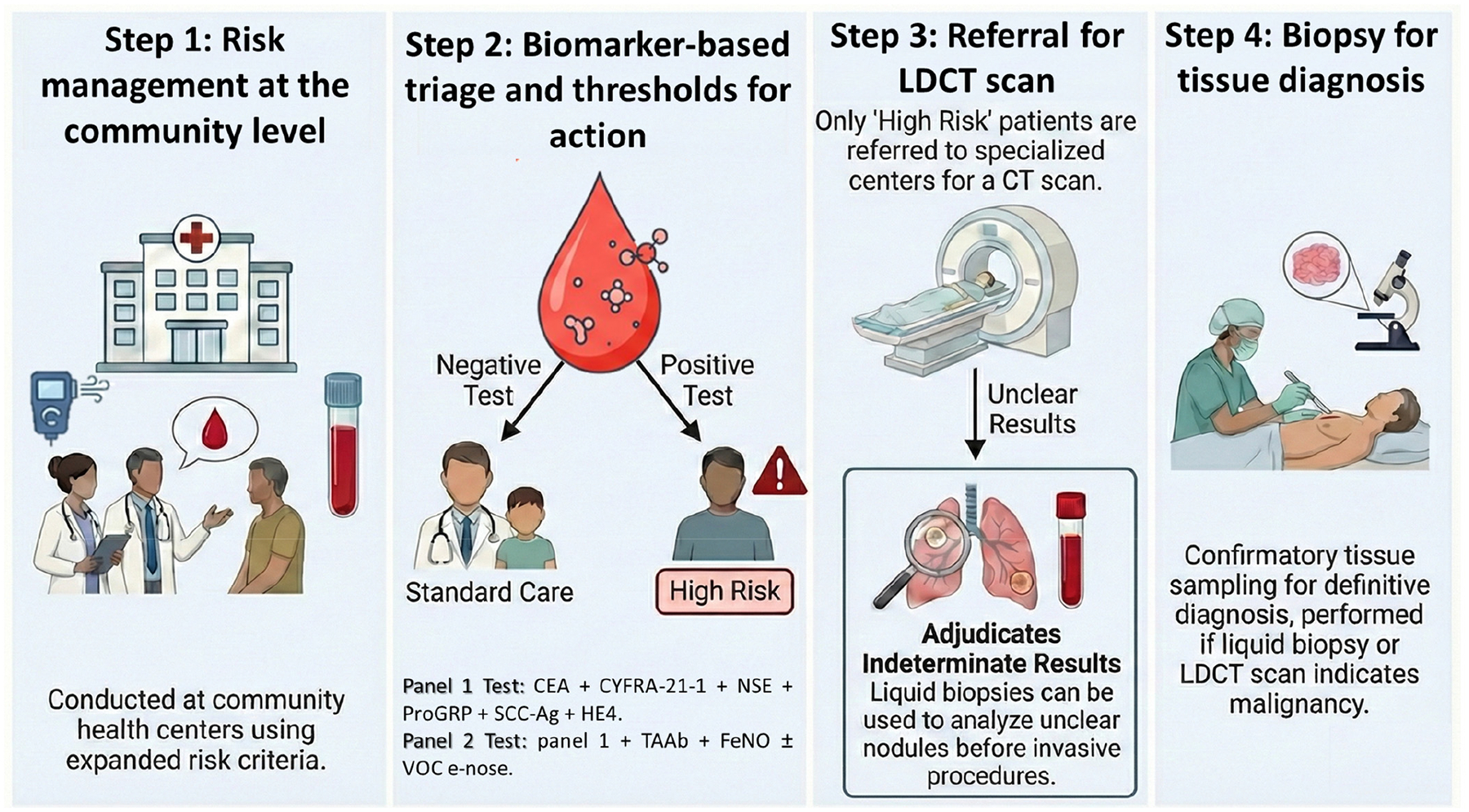
Schematic flowchart of a stepwise biomarker-first triage strategy for lung cancer detection.

Figure 3 outlines a four-step process designed to identify and evaluate at-risk patients, likely for lung cancer.

Step 1—Risk Management at the community level. Primary-care teams in community health centers apply expanded, locally relevant risk criteria (age, tobacco exposure, environmental/occupational factors, and comorbidities) and collect a single blood sample for the core serum biomarker panel (CYFRA 21–1, CEA, SCC-Ag, ProGRP, NSE, HE4), with an optional autoantibody module and point-of-care breath testing when phlebotomy or transport is challenging. The workflow is designed for PR’s distributed care network, as samples can be drawn close to home, transported to a centralized laboratory under standardized procedures, and reported back within a turn-around time that supports timely referral. This step anchors the model in primary care, lowers access barriers for rural municipalities, and aligns testing with payer documentation and authorization needs;

Step 2—Biomarker-based triage and clear thresholds for action. Results from the multianalyte panel are combined with clinical risk to produce a simple, operational decision: “triage-negative” individuals return to routine care and prevention (including smoking cessation and symptom vigilance), while “triage-positive” individuals are flagged as high-risk and prioritized for imaging. The thresholds are calibrated to local prevalence and the capacity to maximize specificity (thereby reducing unnecessary imaging) while maintaining acceptable sensitivity; autoantibody positivity is treated as a rule-in signal. This step is intentionally framed to conserve limited LDCT slots, contain out-of-pocket exposure under capitation models, and make the referral process predictable for primary-care clinicians. For this biomarker-based triage, we propose two types of panels: Panel 1 (minimum viable) is a serum-only panel (CEA + CYFRA-21–1 + NSE + ProGRP + SCC-Ag + HE4) from a single blood draw; Panel 2 (enhanced) adds the TAAb rule-in module and, where feasible, breathomics (FeNO ± VOC “e-nose”) with machine learning integration introduced only after local calibration/validation to reduce overfitting and batch effects. TAAb triage prior to CT has randomized trial support [65];

Step 3—Referral for LDCT scan and adjudication of indeterminate results. Only patients categorized as high-risk are referred to specialized LDCT centers, concentrating imaging resources on those with a higher post-test probability of disease. LDCT reports follow standardized screening nomenclature; when the scan is indeterminate, the pathway introduces a noninvasive liquid-biopsy adjudication (e.g., ctDNA-based or multi-omic signatures) before any invasive procedures are considered. This staged approach reduces avoidable downstream testing, supports patient navigation across island regions, and embeds explicit checks against overuse, which is particularly important where radiology capacity and appointment availability can be constrained;

Step 4—Biopsy for tissue diagnosis. Patients proceed to biopsy when LDCT or the combined imaging-plus-molecular assessment indicates malignancy or high suspicion. Tissue sampling confirms diagnosis and guides definitive management, with a multidisciplinary review to determine the safest, most efficient route (e.g., percutaneous vs. endoscopic). By deferring invasive sampling until after a structured triage and imaging-adjudication sequence, the model minimizes false-positive cascades, reduces patient travel and financial burden, and preserves specialty capacity—fulfilling the manuscript’s goal of a context-adapted pathway for PR that is pragmatic, equitable, and resource-aware.

In PR, this triage-first approach helps to enrich pre-test probability, reduce unnecessary scans/biopsies, and preserve scarce resources. Table 3 shows the advantages of this multi-marker panel approach compared with LDCT.

### Steps for Implementation and Considerations

Effective adoption in PR depends on matching a biomarker-first triage model to day-to-day realities in primary care, radiology, and payment. Below, we outline concise but practical steps.

- Laboratory readiness and quality. Integrated multi-panel biomarker testing should be conducted in primary care (multiplex serum ± TAAb; breathomics where feasible) using pre-specified thresholds tuned to improve specificity while preserving sensitivity. Assays with clear instructions, standardized procedures, and routine quality checks should be used. Test failure/invalid rates should be tracked and decision cutoffs in the local population should be checked before scaling.
  - Baseline risk assessment: age, smoking, clinical factors in primary care
  - Six core serum biomarker panel: CEA + CYFRA-21–1 + NSE + ProGRP + SCC-Ag + HE4 ± TAAb in the same blood draw;
- Logistics and turnaround time. Prioritize sample types and workflows that fit primary care (blood draw or breath analysis). Ensure reliable transport where needed and aim for a turnaround time that supports timely referrals (e.g., within 3–5 business days). Monitor delays and address bottlenecks early;
- Clinical workflow and thresholds. Provide a one-page protocol that states: who is eligible, which biomarker panels are ordered, the exact threshold that triggers LDCT, and when to use liquid biopsy for indeterminate imaging. Include a simple reflex pathway and criteria for multidisciplinary review. All triage-positive cases proceed to LDCT with management governed by Lung-RADS v2022 (American College of Radiology’s structured system for management of findings on LDCT lung cancer screening) to reduce unnecessary procedures and false-positive cascades;
- Fit with capacity and payment. Align test volumes with radiology capacity to avoid overload. Plan the budget across the full pathway (biomarker tests, confirmatory imaging, follow-up visits, patient navigation). Engage payers early to clarify coverage, documentation, and coding;
- Equity and access. Expand access beyond large centers through community clinics, mobile blood collection, or point-of-care breath testing. Provide language-concordant materials and patient navigation to reduce loss to follow-up, especially in rural areas;
- Data and monitoring. Build a simple dashboard to track the time from order to result, invalid rate, proportion of biomarker-positive patients receiving LDCT, number of scans avoided, positive predictive value in practice, and stage at diagnosis. Review these metrics regularly and adjust thresholds if needed;
- Training and communication. Offer brief training for primary care teams on intended use, results interpretation, and next steps. Provide standard referral letters and clear contact channels among primary care, radiology, and oncology;
- Governance and ethics. Ensure appropriate consent, protect privacy, and set a clear plan for communicating incidental or indeterminate findings. Consider community input to maintain trust;
- Phased rollout and pilot. Start with a small pilot in a few clinics and a centralized laboratory. Define success targets (for example, turnaround time ~ 5 days, invalid tests < 5%, fewer unnecessary scans, earlier stage at diagnosis). Use “go/no-go” criteria to decide on expansion and refine the pathway based on real-world results. We will also look for a shift toward earlier stage at diagnosis, higher positive predictive value of the pathway, and shorter time from first presentation to treatment start, with results reported by region to ensure equity.

### Example of Clinical Implementation Scenarios:

The following scenarios illustrate how a biomarker-first triage pathway could operate within PR’s primary-care network and constrained imaging capacity. We propose the following two candidate panels for pragmatic deployment:

Case 1—Primary care-based screening in a screening-eligible smoker. A 62-year-old male residing in a rural municipality presents for routine follow-up in a community primary-care clinic. He has a 35 pack-year smoking history and meets USPSTF eligibility criteria for annual LDCT screening [7]. However, local access to LDCT is limited and requires travel to a tertiary imaging center, which may reduce screening completion. Under a biomarker-first triage workflow, the primary-care team orders the six-marker core serum panel (CEA + CYFRA-21–1 + NSE + ProGRP + SCC-Ag + HE4) proposed in Figure 3 Step 2 (Panel 1)—from a single blood-draw during the visit, with Panel 2 (enhanced) options (TAAb rule-in and/or breath testing), where feasible [65]. Samples are transported to a regional or centralized laboratory under standardized handling procedures with routine quality checks (calibrators/controls, predefined repeat/invalid criteria, and tracking invalid test rates); results are returned within a timeframe that supports timely referrals (e.g., within 3–5 business days, or faster where local capacity allows). Patients classified as triage-negative continue risk reduction and reassessment at defined intervals. Patients classified as triage-positive are prioritized for LDCT referral through a coordinated navigation pathway, with subsequent management governed by Lung-RADS v2022 to reduce unnecessary procedures and false-positive cascades [105]. This approach aims to enrich LDCT referrals, conserve limited imaging slots, and reduce drop-off related to travel and scheduling barriers.

Case 2—Incidental pulmonary nodule risk stratification. A 58-year-old female former smoker undergoes abdominal CT at a regional hospital for unrelated symptoms. Imaging identifies an incidental 6-mm subpleural pulmonary nodule. Standard management often involves serial CT follow-up, contributing to imaging burden and anxiety when pretest malignancy probability is low. In a biomarker-assisted triage model (Figure 3 Step 2), serum biomarker testing (Panel 1) is incorporated as an adjunct to clinical and radiographic risk stratification. A low-risk biomarker profile could support longer surveillance intervals consistent with Fleischner Society guidance for incidental pulmonary nodules [106]. Conversely, a high-risk biomarker signal would prompt earlier LDCT reassessment or advanced imaging evaluation and expedited referral to pulmonology or a multidisciplinary tumor board. When imaging findings remain indeterminate or management uncertainty persists, a methylation-based ctDNA assay can be reserved as an adjudicator (where cost/logistics permit) before invasive procedures are pursued, recognizing that ctDNA/methylation assays are less suited as first-line screening tests in low-prevalence settings [107–110]. This staged approach can reduce unnecessary serial imaging while accelerating the evaluation of higher-risk lesions in settings with constrained radiology capacity. The assay platforms assume that serum proteins and TAAb are measured using standard clinical immunoassay platforms (e.g., automated chemiluminescent immunoassays/CLIA or validated ELISA methods) in a regional or centralized laboratory and that breath testing uses a FeNO device and/or a VOC sensor array (“e-nose”) at the point of care (requiring device calibration and quality checks) [73,111,112].

An operational burden/cost comparison shows that: serum-only testing has the lowest complexity; adding TAAb increases lab complexity modestly; breathomics shifts requirements to point-of-care device calibration and environmental control; and ctDNA/methylation assays are higher cost and more logistically challenging, and are, therefore, best positioned as adjudicators for indeterminate imaging rather than for first-line population screening [108,109,111].

## 5. Conclusions

The global strategies for lung cancer screening face both universal technical hurdles and context-specific implementation barriers. In PR, where socioeconomic and healthcare system challenges are pronounced, relying on the current standard of LDCT is insufficient to address the public health crisis of late-stage lung cancer. Substantially, PR’s low screening uptake reflects multiple restrictions, such as payer and authorization barriers, workforce and imaging-capacity limitations, and participation-related factors such as transportation, health literacy, and fear. A promising and potentially more equitable path forward lies in the validation and implementation of an integrated panel of non-invasive biomarkers (biomarker-first triage strategy). This approach has the potential to transform lung cancer detection on the island, reducing mortality, improving quality of life, and ensuring that access to early diagnosis is a reality for all, not just a privilege for the few.

## Figures and Tables

**Table 1.** Established and emerging technologies for screening of lung cancer.

Test/Marker	Sample Type	Invasiveness	Lung Cancer Subtype(s)	Detection Value
LDCT	Imaging	Low	NSCLC/SCLC (high-risk screening)	Reduces mortality by ~20% vs. chest radiography in randomized trials/meta-analyses.^1^ Sensitivity ~97%, specificity ~87% in screening settings [8,12,17,18,101].
CEA	Blood	Low	NSCLC, SCLC	Useful in combination with CYFRA21–1; improved diagnostic accuracy in case–control/diagnostic cohorts.^2^ Sensitivity ~60–70%, specificity ~70–90% [38,45].
CYFRA21–1	Blood	Low	NSCLC (especially squamous)	Elevated in NSCLC; prognostic and diagnostic utility; used in marker panels.^2^ Sensitivity ~70%, specificity ~90–94%, in diagnostic/three-group case–control [43,45,46].
SCC-Ag	Blood	Low	Squamous NSCLC	Elevated in squamous subtype; best within multi-marker panel with sensitivity >90% per diagnostic case–control^2^ [48–50].
NSE	Blood	Low	SCLC	Elevated in neuroendocrine tumors; aids SCLC differentiation.^2^ Sensitivity ~ 60–75% per diagnostic cohorts [48,51–53].
ProGRP	Blood	Low	SCLC	High sensitivity/specificity for SCLC; higher efficacy with NSE, CEA and CYFRA21–1; best-performing SCLC marker per diagnostic cohort.^2^ Sensitivity 85.7–94.8%, specificity > 90% [48,55–58].
HE4	Blood	Low	SCLC	High AUC in case–control diagnostic studies and high diagnostic accuracy combined with CEA, SCC-Ag, ProGRP and NSE.^2,3^ AUC 0.85–0.99; sensitivity ~64–90%, specificity >96% [47,59–61].
Hp	Blood	Low	NSCLC	Highest detection rate for the squamous NSCLC subtype when combined with CEA, NSE, CYFRA21–1.^2^ Sensitivity ~43–64% but improves substantially in multi-marker panels [62,63].
TAAb	Blood	Low	NSCLC and SCLC	Autoantibodies detectable years prior to diagnosis; population-based RCT evidence shows earlier stage at diagnosis when test-positive individuals receive CT^4^ with high specificity (~90–93%) and modest sensitivity (~30–40%) [64,65].
FeNO	Exhaled Breath	Non	NSCLC	Elevated in lung cancer vs. controls; sensitivity moderate, specificity variable due to inflammatory confounders per diagnostic cohorts [68,69,72].
VOCs/Metals	Exhaled Breath	Non	NSCLC, SCLC	Can differentiate LC from other pulmonary diseases per breath analyses in clinical case–control studies [73,74].
ctDNA	Blood	Low	NSCLC, SCLC	Screening enrichment; indeterminate nodule adjudication when combined with LDCT^5^ in diagnostic and MRD-oriented cohorts [75,77–80].
CTC	Blood	Low	NSCLC, SCLC	Complementary to ctDNA, provides prognostic information and tumor-cell phenotype profiles per diagnostic/monitoring cohort [81].
miRNA	Blood	Low	NSCLC	Early-stage enrichment; screening signal that complements other biomarkers per symptomatic/diagnostic cohorts [83–85].
DNA Methylation (Lavage)	BALF	Low	NSCLC	Outperforms CEA for early-stage detection^5^ per diagnostic cohorts; not suitable for population screening [89].
Near-Infrared Fluorescence Imaging	Tissue	High (Surgical)	NSCLC	Used for intraoperative localization of tumors; not a screening modality [90].

1 Performance values originate from diagnostic or case–control cohorts, except for LDCT, which has randomized trial evidence.

2 Reported sensitivities, specificities, or AUC values correspond to the cited single study unless otherwise noted in the text.

3 HE4 units vary (ng/mL vs. pmol/L) depending on assay manufacturer [59–61].

4 TAAb/EarlyCDT-Lung [64,65]: autoantibody positivity precedes clinical diagnosis; the RCT evaluated triage with CT, not stand-alone screening sensitivity.

5 Performance values for ctDNA and methylation assays are derived from diagnostic or MRD-oriented cohorts, not prospective screening populations. Sensitivity for stage I disease remains limited and reported values may be influenced by clonal hematopoiesis and assay-specific thresholds.

**Table 2.** Detection of different biomarkers in fluids different than plasma for screening of lung cancer.

Fluid Type	Biomarker Name	Detection Metric	Performance Level	Source
Pleural fluid	CEA	89.8% sensitivity, 98.6% specificity (cutoff 5.23 ng/mL)^1^	High	[92]
	CYFRA 21–1	67.9% sensitivity, 90.5% specificity^1^	Moderate	
	SCC-Ag	69.3% sensitivity, 54.1% specificity^1^	Low	
Pleural fluid	NSE	69.0% sensitivity, 45.9% specificity^1^	Low	[92]
	ProGRP	91.7% sensitivity, 97.3% specificity for SCLC^2^	High	[93]
Sputum	CEA	57% sensitivity, 95% specificity^3^	Moderate	[99]
	CYFRA21	36% sensitivity, 95% specificity^3^	Moderate	
	NSE	19% sensitivity, 95% specificity^3^	Low	
Sputum + Plasma	miR-31–5p, miR-210–3p (sputum) and miR-21–5p (plasma)	85.5% sensitivity, 91.7% specificity	High	[85]
Saliva	12-metabolite salivary panel (polyamine/amino-acid pathways)	AUC 0.74–0.79	Moderate	[95]
	let-7a-2, miR-221, miR-20a	95% sensitivity, 85% specificity	High	[94]
	CEA, CYFRA-21–1, NSE, HE4, Hp	N/A^4^	Translational potential/early feasibility	[94,96–98]

1 Pleural fluid performance metrics are from a single cohort and sensitivity/specificity values for each biomarker correspond to that same cohort [92].

2 ProGRP pleural fluid values use the study-specific cutoff from [93].

3 Sputum biomarker metrics are from a single case–control study [99].

4 Metric not reported. This was taken from feasibility studies only. Therefore, no validated cutoffs or diagnostic accuracy are available in the cited papers.

**Table 3.** Comparative analysis: LDCT vs. multi-marker panel for lung cancer detection.

	Global Standard (LDCT)	Integrated Panel
Infrastructure	High (Specialized Centers)	Low (Standard Clinic)
Cost	High (Equipment/Personnel)	Low to Moderate
Accessibility	Urban/Metro Only	Rural Deployable
Invasiveness	Radiation Exposure	Non-Invasive
Performance	Established History	Optimized for Feasibility

## Data Availability

No new data were created or analyzed in this study. Data sharing is not applicable to this article.

## Abbreviations

The following abbreviations are used in this manuscript:

AI: Artificial Intelligence
ALK: Anaplastic Lymphoma Kinase
AUC: Area Under the Curve
ATS: American Thoracic Society
BALF: Bronchoalveolar Lavage Fluid
CEA: Carcinoembryonic Antigen
CT: Computed Tomography
CTCs: Circulating Tumor Cells
ctDNA: Circulating Tumor DNA
CYFRA 21–1: Cytokeratin 19 Fragment
EGFR: Epidermal Growth Factor Receptor
e-nose: Electronic Nose
FeNO: Fractional Exhaled Nitric Oxide
HE4: Human Epididymis Protein 4
Hp: Haptoglobin
IL-17: Interleukin 17
KRAS: Kirsten Rat Sarcoma Viral Oncogene Homolog
LDCT: Low-Dose Computed Tomography
NLST: National Lung Screening Trial
NO: Nitric Oxide
NSE: Neuron-Specific Enolase
NSCLC: Non-Small Cell Lung Cancer
ProGRP: Pro-Gastrin-Releasing Peptide
PPV: Positive Predictive Value
PR: Puerto Rico
ROS1: c-ros Oncogene 1 (Receptor Tyrosine Kinase)
SCC-Ag: Squamous Cell Carcinoma Antigen
SCLC: Small Cell Lung Cancer
TAAb: Tumor-Associated Autoantibody
USPSTF: U.S. Preventive Services Task Force
VOC: Volatile Organic Compound
ECLS: Early Detection of Cancer of the Lung Scotland
Lung-RADS: Lung CT Screening Reporting and Data System
BRFSS: Behavioral Risk Factor Surveillance System
